# A perceptual field test in object experts using gaze-contingent eye tracking

**DOI:** 10.1038/s41598-023-37695-9

**Published:** 2023-07-15

**Authors:** Simen Hagen, Quoc C. Vuong, Liandra Jung, Michael D. Chin, Lisa S. Scott, James W. Tanaka

**Affiliations:** 1 Department of Psychology, University of Victoria, Victoria, Canada; 2 CNRS, CRAN, Université de Lorraine, F-54000 Nancy, France; 3 Biosciences Institute, Newcastle University, Newcastle Upon Tyne, UK; 4 Herbert Wertheim School of Optometry & Vision Science, University of California, Berkeley, USA; 5 Department of Psychology, University of Florida, Gainesville, FL USA

**Keywords:** Psychology, Human behaviour

## Abstract

A hallmark of expert object recognition is rapid and accurate subordinate-category recognition of visually homogenous objects. However, the perceptual strategies by which expert recognition is achieved is less known. The current study investigated whether visual expertise changes observers’ perceptual field (e.g., their ability to use information away from fixation for recognition) for objects in their domain of expertise, using a gaze-contingent eye-tracking paradigm. In the current study, bird experts and novices were presented with two bird images sequentially, and their task was to determine whether the two images were of the same species (e.g., two different song sparrows) or different species (e.g., song sparrow and chipping sparrow). The first *study* bird image was presented in full view. The second *test* bird image was presented fully visible (*full-view*), restricted to a circular window centered on gaze position (*central-view*), or restricted to image regions beyond a circular mask centered on gaze position (*peripheral-view*). While experts and novices did not differ in their eye-movement behavior, experts’ performance on the discrimination task for the fastest responses was less impaired than novices in the peripheral-view condition. Thus, the experts used peripheral information to a greater extent than novices, indicating that the experts have a wider perceptual field to support their speeded subordinate recognition.

## Introduction

An object can be recognized at multiple levels of abstraction. For example, a feathery brown object flitting about in the bush can be categorized as an animal, a bird, a sparrow, or a chipping sparrow. However, one category level, referred to as the basic-level category, has a privileged status in visual object recognition. The basic level captures the optimum amount of perceptual information (e.g., similar global shapes and parts); and as a consequence, objects at this category level bear a perceptual resemblance to one another^1^. Thus, it is argued that the visual input in most cases initially activates a memory representation at the basic level, the so-called entry point of visual recognition^2^. Evidence for basic-level entry comes from category verification studies in which participants are faster to verify that a visual object belongs to a category label at the basic level (*dog*) than at either the superordinate level (*animal*) or subordinate level (*beagle*). The basic-level advantage has been demonstrated across a wide variety of natural and human-made categories^1–4^, as well artificial categories created for laboratory studies^5–7^.

Whereas people generally recognize most object categories at a basic level, those with expertise in a specific domain (e.g., birdwatchers, car aficionados, dog judges) demonstrate a *downward shift* in recognition and recognize objects in their domain of expertise at a more specific level of abstraction (e.g., subordinate level)^4,8^, ^for^ ^reviews^, ^see^^9,10^. Over the last decades, researchers have examined subordinate-level recognition in real-world experts, including experienced bird watchers^4,8,11^, dog show judges^8^, fingerprint specialists^12,13^, and car aficionados^14^. In the laboratory, novices have undergone subordinate level training that promote this downward shift for objects from natural categories (birds)^15,16^, human-made categories (cars)^17^, and artificial categories (Greebles^5,18^, Sheinbugs^19,20^, parametric multipart objects^21,22^, Ziggerins^7^). The results from real-world and laboratory object experts are consistent with the idea that a downward shift in visual recognition occur because of extensive experience individuating visually similar objects^23,24^.

Subsequent research has investigated the diagnostic properties that experts use to facilitate their speeded subordinate level recognition. This work has focused on two properties: color and spatial frequency. Whereas color is a diagnostic property for some basic-level categories (e.g., apple is “red”)^25,26^, experts are more inclined than novices to list color features for subordinate level objects in their domain of expertise (e.g., robin has an “orange” breast)^4^. Hagen et al.^27^ found that experts’ recognition of birds at the subordinate level is disproportionately impaired when color information is removed or altered compared to bird novices. In a follow-up study, bird novices underwent species-level training of naturally colored birds^28^. Following training, the trained novices showed increased sensitivity to bird color, which was also reflected in the N250 ERP component at occipito-temporal channels associated with higher-level visual processes.

Experts also have knowledge of bird shape and parts at a finer grain of detail than novices. For example, bird experts typically name beak shape as a diagnostic feature. The granularity of visual detail in an image can be represented by the spatial frequency (cycles per image [cpi]) in different frequency bands. Whereas low spatial frequencies (in cpi) generally convey coarse-grain level information about the global shape of the object, higher spatial frequencies contain information about finer detail, such as internal part structure^29^. Hagen et al.^30^ masked the external contour of birds and filtered them at different spatial-frequency bands to examine if experts show higher sensitivity to internal parts than novices. They found that both novices and experts were disproportionately more accurate categorizing birds displayed in a middle range of spatial frequencies (8–32 cpi). However, only the experts were also faster categorizing the birds when displayed in this range, indicating an increased sensitivity to the information contained in the middle range of spatial frequencies in experts than novices^30^, ^also^ ^see^^31,32^. These mid-range spatial-frequency bands are also critical for face recognition^33,34^, a form of naturally acquired expertise^35^, indicating that the shape and part information captured by these frequencies are important for other forms of expert subordinate recognition. Overall, these findings indicate that expert recognition is achieved by an increased sensitivity to visual dimensions containing the cues useful for discriminating the subordinate bird categories^4^.

It has been claimed that whereas novices perceive objects in terms of their individual parts, experts see objects in their domain of expertise as unified wholes^e.g.,^^23^. Holistic expert perception has been measured in the composite paradigm where participants are instructed to focus on the top (or bottom) half of an object and to ignore information in the bottom (or top) half. The difficulty of selectively attending to the task-relevant top (or bottom) half of the object, while ignoring the task-irrelevant opposite object half, is interpreted as evidence of a holistic representation that makes it difficult to decouple a whole object into its constituent halves^36^. A composite effect has been shown to depend on real-world expertise, including car experts recognizing car halves^37^, chess experts recognizing chess-board configurations^38^, and in laboratory trained experts recognizing artificial objects^7,39,40^. The holistic percept is thought to be specific to the canonical orientation of the objects. Consistent with the holistic view, the expert recognition of animal experts (dog show judges^41^; Budgerigar experts^42^), expert radiologists^43^ and car experts^44^ is disproportionately impaired when objects in their domain of expertise are turned upside-down. Thus, standard assessments of holistic processing (i.e., composite task, inversion task), indicate that experts recognize their objects of expertise more holistically than novices.

Overall, studies indicate that the fast and accurate subordinate expert recognition is facilitated by increased sensitivity to diagnostic visual dimensions (e.g., color or spatial frequencies) and holistic perception, as defined by an inability to selectively inhibit peripheral object parts in a task irrelevant object half. However, it is unknown if this inability reflect a difference in the ability to perceive information in the periphery away from fixation, or an impairment in the ability to selectively disengage from diagnostic object parts.

### Perceptual fields and object expertise

The field of view where the observer encodes task-relevant visual cues has been referred to as the “perceptual field”^45,46^. Gaze-contingent masking is a technique used to directly test the observer’s perceptual field by systematically manipulating the visual information that is available for any single glance. For example, to assess the perceptual field in face recognition, Van Belle and colleagues^47^ presented faces across three different conditions. First, faces presented in the *central-view* condition restricted the view to one fixated feature (e.g., mouth) using an oval window centered on the gaze position. Second, in the *peripheral-view* condition the oval gaze-contingent window was masked while image regions outside the window were visible (i.e., the non-fixated face features). Finally, in an unrestricted *full-view* control condition, participants viewed the whole image. They found that for recognition of upright faces, accuracy was good and roughly equivalent in the full-view and peripheral-view conditions and recognition in the central-view condition was poor. In contrast, for inverted faces, accuracy was the worst in the peripheral-view condition, but comparable in the full- and central-view conditions. A similar pattern was found for reaction times. Thus, the “non-expert” inverted orientation constricted the perceptual field, consistent with the notion that upright faces are perceived holistically while inverted faces are processed in a feature-by-feature fashion.

Perceptual fields can be influenced by learning and experience. Employing gaze-contingent eye-tracking, studies have shown that expert chess players make better use of peripheral vision to encode a larger span of the chess board than novices^48,49^. Moreover, radiology experts exhibit decreased search times with increasing expansion of the peripheral view^for review, see^^50^. Increased reading skill is associated with a larger perceptual field^51–54^, and more densely packed languages are associated with a smaller perceptual window^55–59^. Some studies report an asymmetry around fixation that depends on the reading direction of the language. For example, readers of left-to-right languages (e.g., English) show a right-biased asymmetry with a larger field to the right compared to left of fixation^59–62^^, for review, see^^63^. Finally, brain injury causing impairments of face recognition (i.e., acquired prosopagnosia) also constricts the perceptual field of face recognition to single face features^64–66^. Across a range of domains with very different visual task requirements, previous work indicates that the size of the observer’s perceptual field expands with learning and experience or expertise.

**Figure 1:**
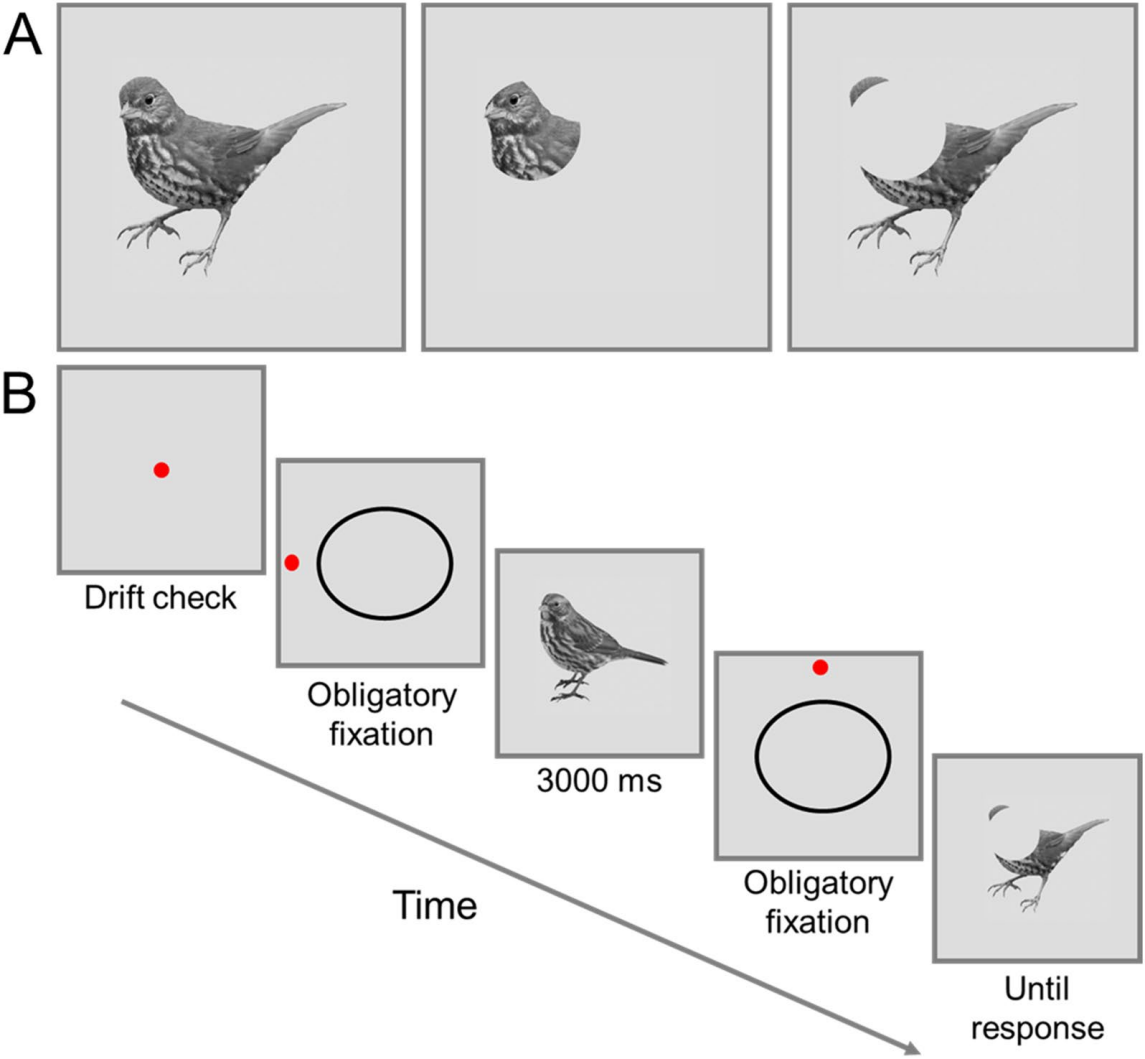
(**A﻿**) an example of the three viewing conditions (full-view [left], central-view [middle], and peripheral-view [right] conditions). (**B**) Example trial. An initial central fixation dot served as a drift check. Next, the participant fixated the “obligatory fixation point” that appeared either left, right, top, or bottom of the ellipse to trigger a bird image to replace the black ellipse. The “study bird” appeared on the screen for 3000 ms, after which the participants once again fixated an “obligatory fixation trigger” next to the ellipse to display the second “test” image. The bird always appeared facing one direction, allowing the participants to prepare saccades to a specific region and the second “test” image was presented randomly in either of the three viewing conditions. This shows an example of a “same” trial where both images display the same bird species.

In the current study, a gaze-contingent paradigm^47,64^ was used to test whether the speeded subordinate-level recognition of the expert is influenced by the visual information that is available in their perceptual field. We selected bird experts because expert bird recognition requires quick, accurate subordinate-level recognition^4,67^. Bird experts and novices were presented with two bird images sequentially, and their task was to determine whether the two images were of the same species (e.g., two different song sparrows) or different species (e.g., song sparrow and chipping sparrow). All images were shown in grayscale to target shape-based expertise processes^30^ and to prevent that the sequential discrimination task was completed by memorizing local color (e.g., red ring around the eye) or global color (e.g., yellow patches around the body and wings) properties. The first *study* bird image was presented in full view. As shown in Fig. 1, the second *test* bird image was presented randomly in either the full-view, central-view or peripheral-view condition. If experts have a wider perceptual field than novices, then the peripheral-view condition would impair experts less than novices. Moreover, if expert recognition depends critically on the peripheral parts, then the central-view condition would impair experts more than novices.

## Methods

### Participants

Fifteen expert participants, ranging in age from 26 to 68 years (7 females,* M* = 46.20 years, *SD* = 16.52 years) were selected based on nominations from their bird-watching peers or from bird watching forums. Fifteen additional age- and education-matched participants who had no prior experience in bird watching, ranging in age from 28 to 66 years (7 females; *M* = 44.40 years, *SD* = 13.22 years), were selected to serve as the novice control group. Power analysis indicated that we had 80% power to detect a between-groups effect of at least Cohen's *d* = 1.06. Nine out of the 15 expert participants, previously participated in our studies on bird recognition^27,30^. Informed consent was obtained from all participants. The study was approved by the University of Victoria Human Research Ethics Office. All methods were carried out in accordance with their guidelines and regulations.

Bird recognition skill-level was assessed with an independent bird recognition test^11,27,30,68^ in which participants judged whether two sequentially presented bird images belonged to the same or different species. In this test, data from one expert was lost due to technical issues, yielding data from 14 experts and 15 novices (this expert was nominated as expert by bird-watching peers, and therefore included in the main analysis). Two (self-nominated) experts recruited from an online forum performed low on this test (*d′* < 0.66, *SE* < 0.43), were removed and replaced by two experts recommended by peers. Thus, while the expert sample size was 15 for the main study, a total of 17 experts were tested all together. Applying a Welch’s two-sample *t*-test to adjust for the unequal sample sizes and unequal variance, we found that the experts obtained a significantly higher discrimination score (*d′* = 1.86, *SE* = 0.14) than the novices (*d′* = 0.87, *SE* = 0.09), *t*(22.42) = 5.95 *p* < 0.001).

### Apparatus

Using a custom MATLAB script (https://github.com/simenhagen/gazeContingent_eyeTracking), stimuli were presented on a 21″ Viewsonic Graphic Series G225f monitor at a viewing distance of 82 cm with a spatial resolution of 1024 × 768 pixels and a refresh rate of 85 Hz. The birds subtended a visual angle of approximately 13.75° horizontally from head to tail. Eye movements were recorded with an SR Research EyeLink 1000 system (SR Research, Osgoode, ON) at a sampling rate of 1000 Hz using a 35 mm lens and a 940 nm infrared illuminator. A chin rest was used to constrain head movements and accuracy of gaze position between 0.25° and 0.50°. Fixations were defined as the period between a saccade onset and offset, using the following parameters for event detection: a motion threshold of 0.0 deg, velocity threshold of 30 deg/s and acceleration threshold of 8000 deg/s^2^.

### Stimuli

The stimuli consisted of different bird species from the Warbler (*n* = 8), Finch (*n* = 8), Sparrow (*n* = 4), and Woodpecker (*n* = 4) families, with each species represented by 12 exemplars for a total of 288 bird images. The stimuli were in part collected from previous studies with experts^11,27,30^, and supplemented with images collected from the Internet. No bird images were repeated in the experiment and therefore each condition consisted of a unique set of bird images. All images were greyscale, cropped and scaled to fit within a frame of 450 × 450 pixels and pasted on a gray background using Adobe Photoshop CS4. All stimuli are available on GitHub (https://github.com/simenhagen/gazeContingent_eyeTracking/tree/main/gc_eyetrack_exp/stimuli_birds_gray). All images were shown in grayscale to target shape-based expertise processes (Hagen et al.^30^) and to prevent that the sequential discrimination task was completed by memorizing local color (e.g., red ring around the eye) or global color (e.g., yellow patches around the body and wings) differences.

### Design

As illustrated in Fig. 1A, a gaze-contingent paradigm was used to create three different viewing-conditions for the second test bird image. In the *full-view* condition, the bird image was fully visible (Fig. 1A, left). In the *central-view* condition, a gaze-contingent circular window was centered on the participants’ gaze position, which restricted their view to the central region of the visual field while masking the peripheral region (Fig. 1A, middle). In the *peripheral-view* condition, a gaze-contingent circular mask was centered on participants’ gaze position, which masked the central region while allowing the peripheral region of the visual field to be visible (Fig. 1A, right). The window and mask subtended 5.81° horizontally and 5.17° vertically of visual angle (pixel diameter = 190).

Unlike previous studies^47,64^, the size of the window and mask was determined in a pilot study with a different group of novice participants to find the size that yielded approximately equal performance in the *full-view* and *central-view* conditions and a substantial impairment in the *peripheral-view* condition. The rationale was that this size would approximate the spatial range from which cues are perceived by novices and to which experts can be compared. This approach was taken since bird parts are challenging to define and have different sizes (e.g., small beak compared to large wing-pattern), thereby preventing a window size that contained single object parts (as possible for facial parts).

### Procedure

Participants were tested in a sequential same-different matching task while their gaze positions were monitored. They were shown a sequence of two bird images and instructed to respond “same” (“c” on the keyboard) if the bird images were of the same species or respond “different” (“m” on the keyboard) if the bird images were of different species. For the *same* trials, the birds were different images of the same species (e.g., two field sparrows), and for the *different* trials, the birds were images of different species from the same family (e.g., field sparrow versus a song sparrow). The participants were instructed to respond as quickly and accurately as possible.

As illustrated in Fig. 1B, each trial began with a red fixation dot at the center of the screen that served as a drift check, by measuring deviations relative to calibration. Large deviations (i.e., > 2.0°) prompted recalibration. Acceptable drift deviations were followed by a new red fixation dot that appeared either to the left, right, above, or below a centered black oval shape (16.16 deg. horizontally from the center point of the screen). The location of this red dot was randomly determined on each trial. The oval shape served as a cue to where the bird would appear. Once participants fixated on the red dot (i.e., a fixation was registered in a small window surrounding the dot), the first *study* bird image was presented in full view and remained on the screen for 3000 ms. It was then replaced by another black oval shape paired with a red fixation dot that appeared randomly on either of its sides, or above or below. Again, once participants fixated on the red dot, the second *test* bird image was randomly presented in either of the three viewing conditions until a manual (button) response was made. This procedure ensured that every participant fixated off the bird before it appeared on the screen. The participants were also informed that the three viewing conditions would appear at random with an equal probability, and that the birds would always be presented with the head in the same left facing direction.

There were 48 trials (24 *same* trials, 24 *different* trials) each for the *full-view*, *central-view*, and *peripheral-view* conditions for a total of 144 trials. Trials from the two trial types and three viewing conditions were presented in a random order, to prevent participants from adopting any strategies for the different viewing conditions. In addition, participants completed 6 practice trials with images not used during the experimental phase.

### Data analysis

Our primary analysis of interest for the gaze-contingent paradigm was the effect of expertise and viewing condition on recognition performance when participants were presented with the *test* bird image. The performance measures included sensitivity (*d′*) and correct response times (RTs). Following our previous work^27,30^, we also analyzed sensitivity for different RT bins to test whether viewing conditions differentially affected experts and novices in the fastest and slowest responses.

We also conducted secondary analyses for the eye-tracking data during the presentation of the *study* bird image. Eye-tracking data from one expert was lost due to a technical error, yielding eye-tracking data for 14 experts and 15 novices (in contrast to behavioral data for 15 experts and 15 novices). For the results, we present the viewing patterns first, followed by our primary analyses of interests. In the SI, we present additional analyses for the *test* image related to fixation count, fixation duration, etc., for completeness.

### Transparency and openness

The study was not preregistered. The experimental code and stimuli can be found on GitHub (link provided above).

## Results

### Eye movements during the study bird

**Figure 2:**
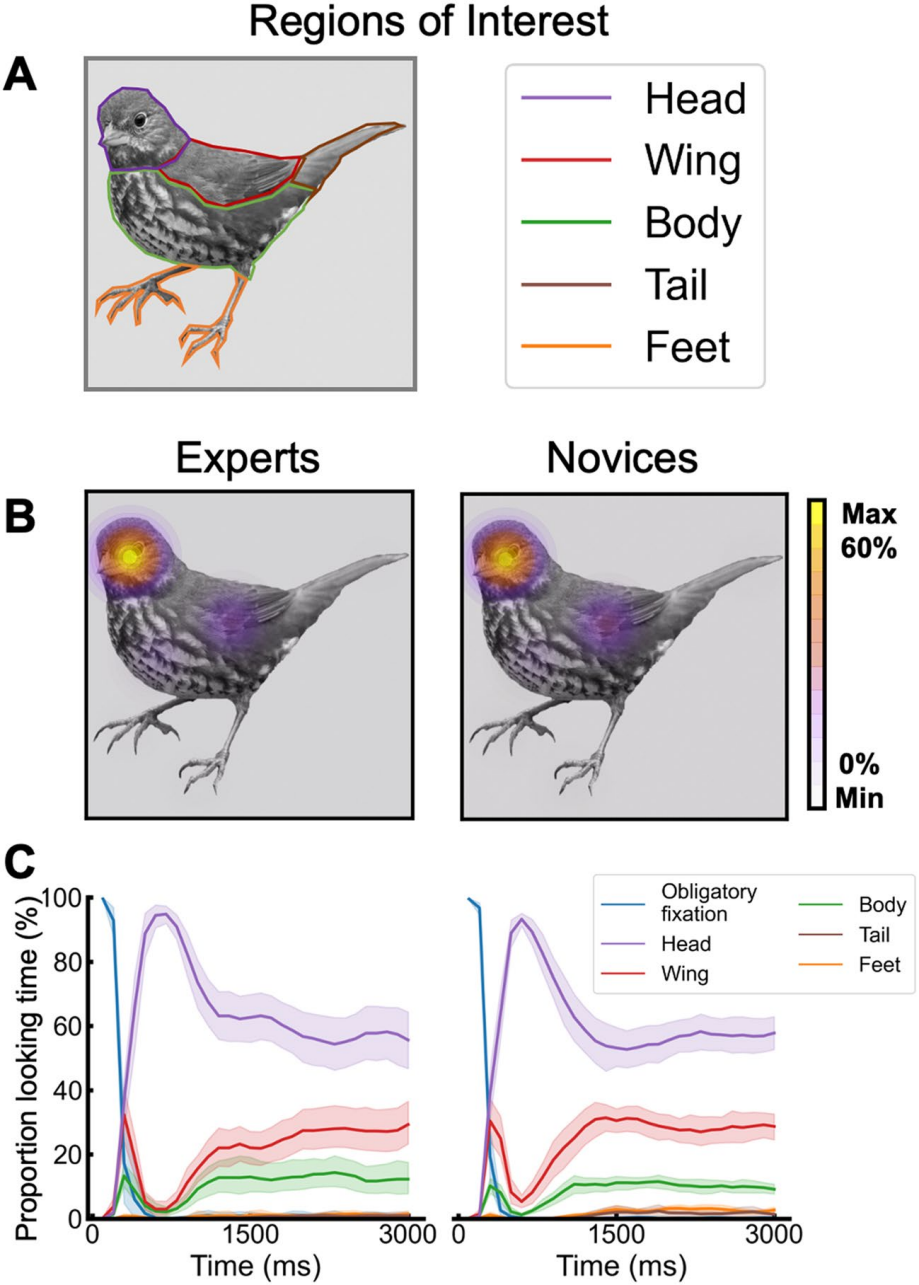
Eye-tracking analysis for the “study” bird. (**A**) Five bird ROIs that were manually drawn on each bird image (head, wing, body, tail, and feet). (**B**) Average percentage of looking time as a function of ROI for experts (left) and novices (right). (**C**) Temporal unfolding of proportion looking time as a function of time relative to the stimulus onset for experts (left) and novices (right). Within each time window of 100 ms (30 time-windows), the fixation duration in each ROI was divided by the total fixation duration in that window. Obligatory fixation ROI is included here to illustrate that all participants started fixating the same point outside the bird. Error bars represents SEMs.

*Defining bird regions of interests (ROIs)*. Five regions of interest (ROIs) were manually drawn on each bird image, corresponding with the bird’s head, wings, body, tail, and feet. Figure 2A illustrates these ROIs for an exemplar bird image. Any fixations outside of the bird (i.e., not in any ROI) were excluded from further analyses. Proportion looking time was computed for each ROI as the time fixated in each ROI divided by the total fixation duration across all five ROIs (i.e., the whole bird).

### Average fixation duration by ROI

Figure 2B presents mean proportion fixation duration as a function of group (experts, novices) and ROI (head, wings, body, tail, feet). The fixation duration within each ROI was divided by the total fixation duration across all ROIs (i.e., only including fixations within the bird) separately for each participant. The fixation data was analyzed in a 2 × 5 mixed design ANOVA with group as a between-subjects factor and ROI as a within-subjects factor. The main effect of group was not significant, *F*(1, 27) < 1.0. The significant main effect of ROI, *F*(4, 108) = 253.95, *p* < 0.001, generalized eta^2^ = 0.90, showed that fixation across both groups were largely at the head ROI (*M* = 44.28%; head vs. all other regions, all *p*s < 0.001) followed by wings (*M* = 23.24%; wings vs. all remaining ROIs, *p*s < 0.001) and body (*M* = 17.05%; body vs. all remaining ROIs, all *p*s < 0.001). Fixations in the feet and tail ROIs did not differ (*M*_*feet*_ = 7.79%; *M*_*tail*_ = 7.65%; *p* = 0.864). Group and ROI did not interact, *F*(4, 108) = 0.482, *p* = 0.749.

### Time course of viewing times by ROI

Figure 2C shows the temporal unfolding of fixations across ROIs separately for experts and novices, by extracting 100 ms time windows relative to stimulus onset and computing within each time window the proportion of viewing time in each ROI (ROI fixation duration / total fixation duration within the bird in that time window). There was a strong correlation between the experts’ and novices’ temporal unfolding of viewing time for each ROI (e.g., the head ROI temporal trajectory for experts correlated strongly with that of novices’) (all ROIs, *r*s > 0.86, all *p*s < 0.001). For illustrative purposes, we also plotted the time course corresponding to the obligatory fixation point that “triggered” the bird image.

### Manual responses to the test bird

Next, we analyzed the manual response data, and the corresponding eye-tracking data for the *test* image (second bird image), which was subject to the gaze-contingent manipulation. This was response contingent with eye-tracking terminated upon the manual response. The main aim was to examine recognition performance as a function of viewing condition (full-view, central-view, peripheral-view) and group (expert, novice). Note that the size of the window/mask applied in the central and peripheral view conditions was calibrated through pilot testing to approximate the perceptual window of novices. The rationale was that if experts perceived the birds holistically, then their recognition should be less impaired by masking central view.

### Sensitivity analysis for manual responses

**Figure 3:**
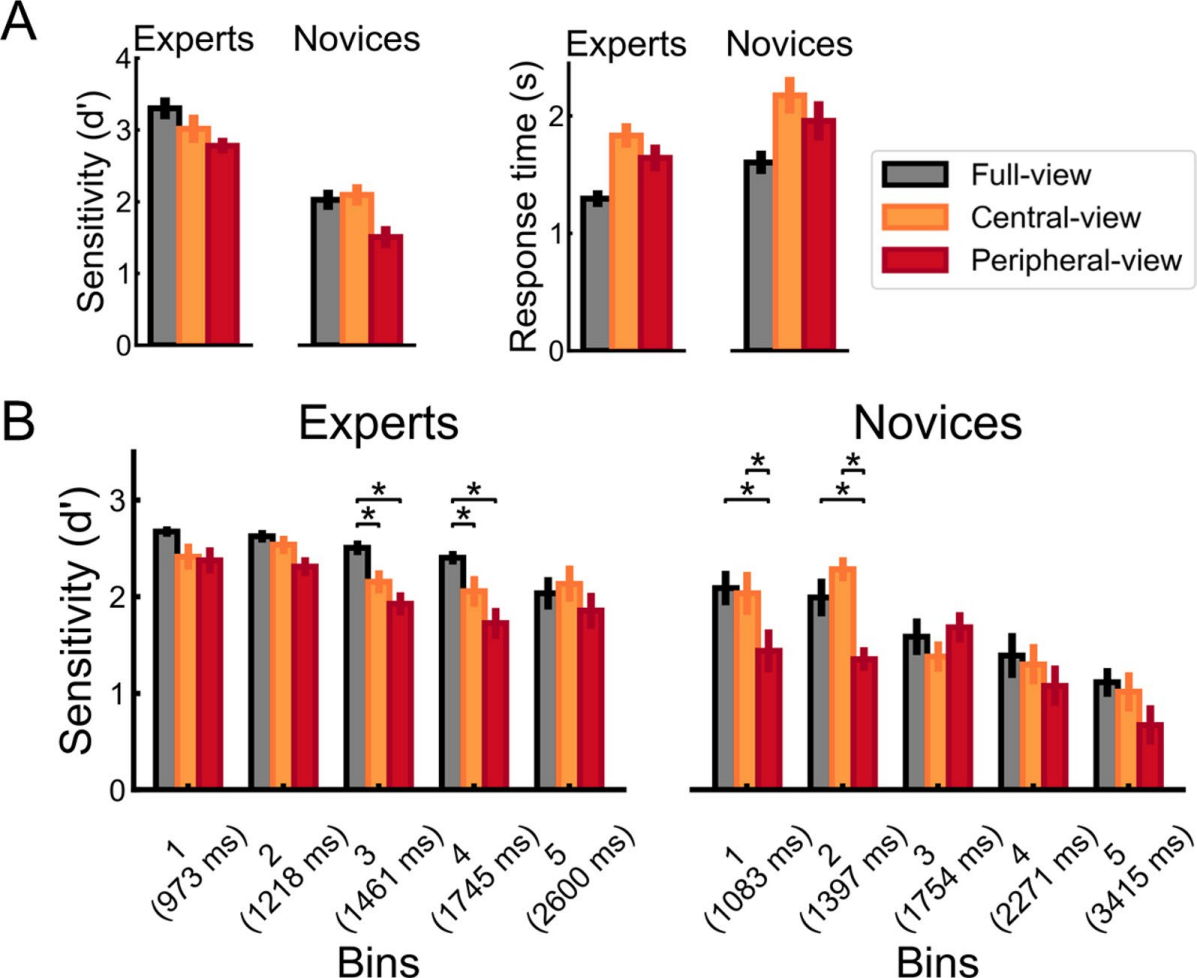
(**A**) d’ (left) and correct RT (right) as a function of group (experts, novices) and viewing condition (full-view, central-view, peripheral-view). (**B**) Distribution of d’ scores for the experts (left) and the novices (right). Bin 1 contains the 20% fastest responses of each participant. Bin 2 contains the next 20% fastest responses, and so on. Average response times for each bin is represented in brackets on the x-axis. Error bars represent SEMs.

Trials with RT 3 SD (1.92% of total trials) greater than each participant’s grand mean was excluded from this and all subsequent analyses. Figure 3A (left) presents mean d’ scores as a function of viewing condition (full-view, central-view, peripheral-view) and group (experts, novices) (see SI for ACC data). For this study, hits were defined as responding “same” on *same* trials, and false alarms were defined as responding “same” on *different* trials. The sensitivity measure (*d′*) was computed as: *Z*(hit rate) – *Z*(false-alarm rate), with hit rate calculated as hits + 0.5/(hits + misses + 1) and false alarm rate as false alarms + 0.5 / (false alarms + correct rejections + 1)^69,70^. The d’ data were analyzed in a 2 × 3 mixed design ANOVA with group (expert, novice) as the between-subjects factor and viewing condition (full-view, central-view, peripheral-view) as the within-subject factors. The significant main effect of group, *F*(1, 28) = 79.46, *p* < 0.001, generalized eta^2^ = 0.60, showed that experts were better at discrimination of the birds relative to the novices (novices: *M* = 1.70, *SE* = 0.10; experts: *M* = 3.03, *SE* = 0.11). The significant main effect of viewing condition, *F*(2, 56) = 8.79 , *p* < 0.001, generalized eta^2^ = 0.13 (full: *M* = 2.57, *SE* = 0.11; central: *M* = 2.46, *SE* = 0.14; peripheral: *M* = 2.07, *SE* = 0.10), showed that sensitivity in the full-view and central-view was higher than in the peripheral-view (all *p*s < 0.005), while the sensitivity in the full- and central-view conditions did not differ (*p* = 0.438). Group and viewing condition did not interact, *F*(2, 56) = 1.15, *p* = 0.326.

### Response times for correct manual responses

Figure 3A (right) presents the mean correct RTs as a function of group (experts, novices) and viewing condition (full-view, central-view, peripheral-view). The RT data were analyzed in 2 × 3 mixed design ANOVA with group (expert, novice) as the between-subjects factor and viewing condition (full-view, central-view, peripheral-view) as the within-subject factors. The main effect of group approached significance, *F*(1, 28) = 3.91, *p* = 0.058, generalized eta^2^ = 0.11). The significant main effect of viewing condition, *F*(2, 56) = 54.50, *p* < 0.001, generalized eta^2^ = 0.20 (full: *M* = 1449 ms, *SE* = 60 ms; central: *M* = 2004 ms, *SE* = 102 ms; peripheral: *M* = 1799 ms, *SE* = 107 ms), showed that correct response times were faster in full-view than peripheral-view and central-view (*p*s < 0.001), and that central-view was slower than peripheral-view (*p* = 0.001). As with the sensitivity analysis, group and viewing condition did not interact, *F*(2, 56) = 0.05, *p* = 0.947.

### Response time distribution analysis

Next, we examined how viewing condition affected expert and novice recognition during their fastest and slower reaction times. This analysis was motivated by the reasoning that faster trials reflect to a larger degree automatic responses than slower trials, and that a hallmark of expertise is rapid and automatic recognition^e.g.,^^22,23,71^. Indeed, we previously showed that experts and novices differed in their sensitivity to color and spatial-frequency information during their fastest responses^27,30^.

We analyzed d’ scores as a function of response speed. Specifically, each participant’s trials were sorted from fastest to slowest separately for each viewing condition and trial type. Next, the trials were grouped into five bins containing both the fastest 20% of responses from *same* trials and the fastest 20% of responses from *different* trials (i.e., quintile bin 1), the next 20% of responses from both trial types (i.e., quartile bin 2), and so on. Within each bin, mean d’ scores for each condition for each participant were computed.

Figure 3B presents mean *d’* as a function of group (experts, novices), viewing condition (full-view, central-view, peripheral-view) and quintile bin (1, 2, 3, 4, 5). The data were first analyzed in a mixed-design ANOVA using viewing condition and bin as within-subjects factors, and group as a between-subjects factor. The main effects of group, *F*(1, 28) = 51.29, *p* < 0.001, generalized eta^2^ = 0.28, bin, *F*(4, 112) = 27.70, *p* < 0.001, generalized eta^2^ = 0.18, and viewing condition, *F*(2, 56) = 15.92, *p* < 0.001, generalized eta^2^ = 0.07, were significant. Viewing condition did not interact with group, *F*(2, 56) = 0.7**,** *p* = 0.502, or bin, *F*(8, 224) = 1.37, *p* = 0.21. In contrast, group interacted with bin, *F*(4, 112) = 2.67, *p* = 0.036, generalized eta^2^ = 0.02, and crucially, the three-way interaction between group, bin, and viewing condition was significant, *F*(8, 224) = 2.06, *p* = 0.041, generalized eta^2^ = 0.03 (see also SI for group x bin x viewing condition interaction in the accuracy data).

Given the three-way interaction, we examined the effect of viewing condition on group separately for each bin. In Bins 2 and 3, the two-way interaction between group and viewing condition was significant, *F*(2, 56) = 3.29, 3.35, *p* = 0.005, 0.042, generalized eta^2^ = 0.07, 0.06, respectively This interaction was marginally significant in Bin 1, *F*(2, 56) = 2.58, *p* = 0.085, generalized eta^2^ = 0.04. We accepted this interaction at the one-tailed level given that our previous research indicated a general pattern of differences between experts and novices for fast responses (Hagen et al.^27,30^; see also SI for group x viewing condition interaction for these bins in the accuracy data). A separate ANOVA per group within each Bin (1, 2) revealed a significant effect of the viewing condition for the novices, but not the experts (Novices: all *F*s > 6.79, *p*s < 0.004, all general eta^2^ > 0.14; Experts: all *F*s < 2.34, *p*s > 0.115). Post-hoc paired t-tests showed that the novices had higher d’ in the full-view and the central-view than the peripheral-view (Bins 1 and 2: uncorrected *p*s < 0.018), while full-view did not differ from central-view (Bins 1 and 2: uncorrected *p*s > 0.193). In contrast, a separate ANOVA per group within Bin 3 revealed a significant effect of the viewing condition for the experts, but not the novices (Experts: *F*(2, 28) = 7.0, *p* = 0.003, generalized eta^2^ = 0.22; Novices: *F*(2, 28) = 0.94, *p* = 0.403). Post-hoc tests showed higher d’ for the experts in the full-view than the central-view (uncorrected *p* = 0.022) and the peripheral-view (uncorrected *p* = 0.003), while recognition did not differ in the central-view and the peripheral-view (uncorrected *p* = 0.199). Finally, in Bins 4 and 5, the two-way interaction between group and viewing condition was not significant (Bins 4 and 5: all *F*s < 1.0, *p*s > 0.526).

Separate analysis presented in the SI confirmed that the expert peripheral-view advantage was not explained by a speed-accuracy trade-off, nor did novices’ accuracy in the peripheral-view condition increase with longer RTs (e.g., to strategically shift attention to the periphery). Moreover, the advantage was not explained by differences in average fixation duration (e.g., longer fixations to divert attention away from fixations; SI). Finally, the viewing condition did not differentially impair recognition in experts and novices in terms of average fixation durations or fixation rate (see SI).

In summary, the gaze patterns during free-view (study image) of the experts and novices were strikingly similar (see SI for Bayes factor analysis). However, while the gaze-contingent central-view did not differentially impair the recognition of the experts and novices, the gaze-contingent peripheral-view impaired the recognition of experts less than novices for the fast responses. Thus, while the novices used largely central-view information, the experts used both central- and peripheral-view information for speeded recognition.

## Discussion

The aim of this study was to examine whether real-word expert object recognition changes the perceptual field for objects in the domain of expertise. Using gaze-contingent eye tracking and a discrimination task, bird experts and age-matched novice participants made “same/different” within-species (i.e., subordinate category) judgements to sequentially presented pairs of bird images. The first *study* image was always presented in full view, and the second *test* image was presented randomly in either a full-view, central-view or peripheral-view condition. If experts have a larger perceptual field or processed information differently in the field than novices, then the bird experts’ discrimination performance would be less impaired than the novice’s performance in the peripheral-view condition. Moreover, the degree to which the peripheral information is critical to their recognition would be reflected in the interference caused by the central-view condition.

Overall, the results showed that the experts discriminated the birds more quickly and accurately than novices, consistent with previous work^4,27,30^. While the overall analysis showed no difference between experts and novices as a function of viewing condition, group differences emerged in the quintile distribution analyses in which gaze-contingent effects were examined as a function of recognition speed. These analyses showed that the peripheral-view condition disrupted recognition relative to the full- and central-view conditions for the novices but not for the experts in the fast trials (Bins 1 and 2). Moreover, the central-view condition generally showed comparable sensitivity performance to the full-view condition for both groups in most quintile bins. Thus, during speeded recognition, the experts recognized the birds using peripheral information better than the novices, but their recognition did not decline when limiting the view to information only in central view. We used a one-tailed significance level for the fastest responses (Bin 1) as the current findings are in line with our previous work using similar distribution analyses^27,30^. Furthermore, control analyses ruled out alternative explanations including speed-accuracy trade-offs and differences in single fixation durations (see SI for details).

These findings are consistent with studies reporting that expertise influence the width of the perceptual field in other domains of expertise, including chess, radiology, reading, and face recognition (as discussed in the introduction). Within all of these domains, expertise is associated with better use of peripheral vision to perceive task-relevant information. The current results, combined with the previous work, suggest that widening of the perceptual field size is a general visual learning phenomenon that cuts across a range of domains with different task demands (e.g., visual search in radiology vs. object categorization in bird watching). The development of a wider perceptual field could result from the need to rapidly and accurately detect and recognize complex task-relevant cues within a visual domain. With regard to object expertise, future work using in-lab training paradigms could test how subordinate discrimination experience with homogenous object domain influence the perceptual field size or how visual information is processed in the perceptual field.

The expert peripheral advantage in the fast responses suggest that the experts utilize a wide perceptual field, whereby both central and peripheral information is available, specifically for birds that are rapidly recognized. In contrast, the lack of expert peripheral advantage in the relatively slower responses, indicate that the experts use a more focused strategy in which local cues are attended to a larger degree for birds that are recognized more slowly. Previous studies analyzing response time distributions also show expert-novice differences during fast responses. For example, bird experts use object color for family-level recognition in both fast and slow responses, while novices use it only for slower responses^27^. Moreover, bird experts use a middle range of spatial frequencies in fast and slow family-level recognition, while novices show no spatial-frequency advantage in fast or slow trials^30^. Collectively, these studies suggest that different perceptual strategies are employed by experts depending on whether recognition is fast or slow, with fast recognition instances deviating the most from novice recognition. One possibility is that fast expert recognition reflects the subcategories for which the expert has the most refined knowledge of diagnostic object parts and colors (beak, wings, breast of a bird), allowing the retinal input to activate the object memory despite blocking a subset of the diagnostic information in the central-view condition in the current study.

How does the current results relate to previous reports of holistic expert recognition? While the composite effect for experts show that they find it difficult to ignore irrelevant object parts^37^, this effect could reflect stronger part binding for experts than novices *within* an equally sized perceptual field. In other words, the experts could automatically select multiple features, while novices selectively focus on single/fewer features, within an equally sized perceptual field. Our design allowed us to test whether experts and novices have a different perceptual field size *independent* of being tasked to suppress task-irrelevant object cues. Thus, the observation that experts use peripheral cues for rapid recognition to a larger extent than novices add to the previous reports of holistic recognition using the composite effect: Experts show both holistic recognition (previous studies) and a wider perceptual field (current study), while novices show non/less-holistic recognition (previous studies) and narrower perceptual field (current study). Future studies on real-world object recognition can compare composite and inversion paradigms with gaze-contingent eye-tracking to examine if similar processes underlie holistic perception and changes to perceptual fields.

In contrast to the expert and novice differences we report for the viewing condition, we found no differences between the groups when examining their fixations to different bird regions during the presentation of the *study* image in full view. Specifically, both groups fixated the same bird regions, with most of their fixations in the head, wing and chest regions, respectively. Moreover, the temporal unfolding of their fixations did not differ, with the initial fixation mostly in the head region. Similar analyses of the *test* image showed identical patterns. However, supplementary analysis of the fixation behavior to the test image revealed that experts and novices differed to some extent in the last fixation point before making a response (see SI). Thus, while the overall gaze behavior is strikingly similar, there can be subtle differences that can be investigated in future work.

The lack of substantial difference in eye movements between experts and novices is consistent with studies of face recognition that report no differences for conditions that preserve expertise versus those that do not. For example, for naturally acquired expertise^35,72^, upright and inverted faces show similar eye-movements^47,73^, as do prosopagnosics and controls^65,66^, but see^74^. In contrast, for studies on chess expertise, expert chess players display fewer fixations and have more fixations between pieces than less experienced players during recognition of chess configurations^48,49,75,76^. Similarly, expert radiologists have longer saccades and fewer fixations than less experienced observers while searching for tissue abnormalities in x-rays^77–80^. A recent study also showed that naïve participants who learn to categorize novel objects at a subordinate level exhibit an increase in average fixation duration and saccadic amplitude pre- to post-training^20^. It is possible that in our current task, perceptually salient object regions overlap with regions that are diagnostic for recognition, thereby masking eye-movement differences between experts and novices. Moreover, eye-movement differences are likely to be observed between bird experts and novices if they were asked to search for the birds in a visual scene, consistent with findings showing that car detection in visual scenes correlate strongly with car expertise^81^, although this may depend on the distractor category used^82–84^. Importantly, the current study shows that the gaze-contingent effect appears despite highly similar overall eye-movement behavior.

In summary, we found that bird experts can recognize birds using visual information relatively far away from central fixation compared to non-experts. This is consistent with findings from other visual expertise domains, where expertise is associated with a relatively wide perceptual field (as discussed in the introduction). While the lack of substantial differences in eye movements suggest that domain expertise depends on how a retinal input is processed, such null results should be interpreted with caution, as perhaps a more sensitive paradigm and analysis could result in differences between experts and novices. We focused on shape processing in the current study. Future work can investigate if surface color modulate how experts process peripheral information, given past reports of experts’ sensitivity to color information^27^. Moreover, future work can examine how expert recognition relates to spatial processing in the human ventral-occipito-temporal cortex^85^, neural sensitivity to different object parts and color patches^86^, and sensitivity to whole birds presented beyond central vision^87^.

## Supplementary Information

Supplementary Information.

## Data Availability

The data can be requested upon emailing the corresponding author.
