# Supplementary material for: A perceptual field test in object experts using gaze-contingent eye tracking
Source: Sci Rep. 2023 Jul 15;13:11437. doi: 10.1038/s41598-023-37695-9 (PMC10349839; doi:10.1038/s41598-023-37695-9)
Supplement: Supplementary file 1 — Supplementary Information. [file 41598_2023_37695_MOESM1_ESM.docx]

SI for *A perceptual field test in object experts using gaze-contingent eye tracking*

**Bayes factor analysis**

For the eye-tracking analysis to the “study” image, given the null findings for a group by ROI effect, and a group by ROI x time point effect, we conducted Bayes factor analysis to examine if the evidence favored the hypothesis that fixation durations were equal for the two groups as a function of ROI and as a function of ROI and time point. We used a repeated-measures Bayes analysis (group × ROI x time points) implemented in the BayesFactor package in the R programming language^1^ with its default settings for priors (models, priors, and methods of computation are provided in Rouder et al.^2^). We followed the interpretation of Bayes factors of Jeffreys^3^ as adopted by Wagenmakers et al.^4^ and accepted Bayes factors > 3 as an indication for differences in performance, Bayes factors < 1/3 as an indication for consistent performance, and Bayes factors between 1/3 and 3 as anecdotal evidence for differences in performance.
 The Bayes analysis was in line with the ANOVA. There was no evidence (i.e., consistent fixation duration) for an interaction between Group and ROI (Bayes factor = .12), nor for an interaction between group, ROI, and time point (Bayes factor < .001). In summary, there was no evidence for differences between experts and novices in the ROI analysis.

**Quintile analysis**

*Distribution analysis for correct reaction times.* Here we run a response time distribution analysis with the correct reaction time data, to test for speed-accuracy trade-offs in the similar analysis we run with the d’ data. Figure S1A presents mean correct reaction times as a function of quintile bin (1, 2, 3, 4, 5), viewing condition (full-view, central-view, peripheral-view), and group (experts, novices). A mixed-design ANOVA showed no significant interaction between group, viewing condition, and bin, *F*(8,224) = 0.82, *p* = 0.583. This finding suggests that differences between the experts and novices in each bin for the primary d’ analysis was not due to a speed-accuracy trade-off.

Analyses targeting Bins 1, 2, and 3, further showed that the difference between the experts and novices in terms of d’ and accuracy are not due to speed-accuracy trade-offs. Separately for each bin, a mixed-design ANOVA with the overall response times in the bin using viewing condition as a within-subjects variable and group as a between-subjects, showed no significant interaction for Bins 1, 2, and 3 between group and viewing condition**,** *F*(2,56) = 1.53, 1.21, 0.50, *p* = 0.225, 0.306, 0.610, respectively. Thus, the response times associated with the d’/accuracy in Bins 1, 2, and 3 were not differentially affected between experts and novices as a function of viewing condition.


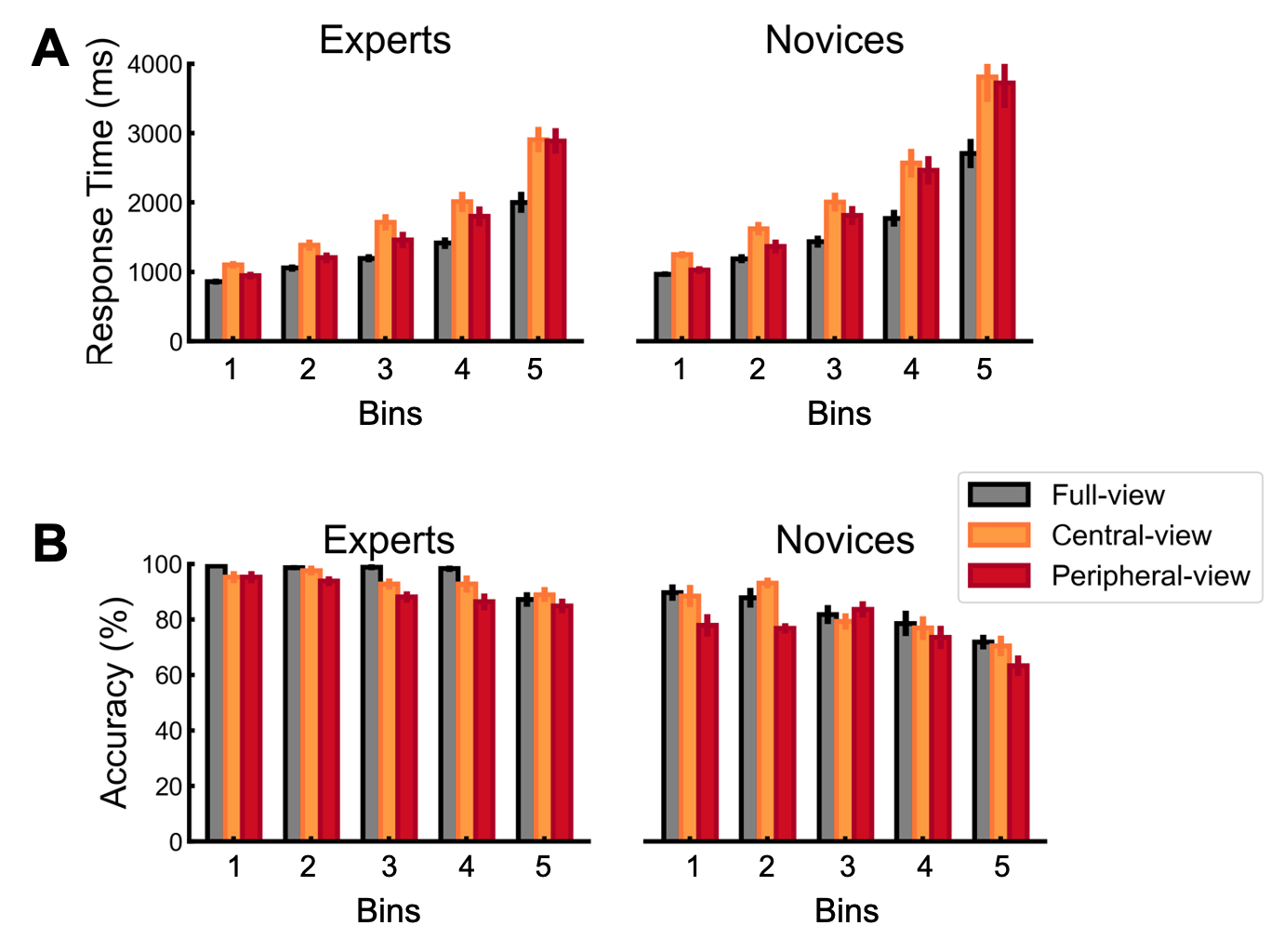


***Figure S1.*** *Quintile analysis of overall manual responses.* ***A.*** *Correct RTs as a function of quintile bin for the experts and the novices.* ***B.*** *Accuracy as a function of quintile bin for the experts and the novices. Bin 1 contains the 25% fastest responses of each participant. Bin 2 contains the next 25% fastest responses, and so on. Error bars represent the SEMs.*

*Distribution analysis for accuracy*. Here we run a distribution analysis with the accuracy data, to complement the distribution analysis of the d’ data. Figure S1B presents mean accuracy as a function of quintile bin (1, 2, 3, 4, 5), viewing condition (full-view, central-view, peripheral-view), and group (experts, novices). The data were first analyzed in a mixed-design ANOVA using viewing condition and bin as within-subjects factors, and group as a between-subjects factor. The main effects of group, *F*(1, 28) = 48.61 , *p* < 0.001, generalized eta^2^ = 0.28, bin, *F*(4, 112) = 23.65 , *p* < 0.001, generalized eta^2^ = 0.17, and viewing condition, *F*(2, 56) = 14.15 , *p* < 0.001, generalized eta^2^ = 0.07, were significant. Group did not interact with bin, *F*(4, 112) = 2.23 , *p* = 0.07. Group did not interact with viewing condition, *F*(2, 56) = 0.7, *p* = 0.5. Moreover, bin did not interact with viewing condition, *F*(8, 224) = 1.14 , *p* = 0.336.

The three-way interaction between group, bin, and viewing condition was significant, *F*(8, 224) = 2.25, *p* = 0.025, generalized eta^2^ = 0.03. We therefore examined the group by viewing condition effect in each bin using a mixed-design ANOVA with viewing condition as a within-subjects factor, and group as a between-subjects factor. Here we only report the critical group by viewing condition effects. In Bins 1, 2, and 3 the significant two-way interaction between group and viewing condition, *F*(2, 56) = 3.46, 3.30, 3.53, *p* = 0.038, 0.044, 0.036, generalized eta^2^ = 0.05, 0.07, 0.06, respectively, indicated that viewing condition had a differential effect on the experts and the novices.

In Bin 1, for the experts, the peripheral-view, but not the central-view differed from the full-view (*p* = 0.04, 0.111, respectively). No difference was found between central- and peripheral-views (*p* > .999). For the novices, the peripheral-view differed from full- and central-views (*p* = 0.005, 0.018, respectively), while central- and full-view did not differ (*p* = 0.675). In Bin 2, for the experts, the central-view did not differ from full- or peripheral-views (*p* = 0.638, 0.225, respectively), nor did peripheral- and full-view differ (*p* = 0.117). For the novices, the peripheral-view differed from full- and central-views (*p* = 0.021, 0.002, respectively), while central- and full-view did not differ (*p* = 0.183). Thus, for Bins 1 and 2, the novices were disproportionately impaired by peripheral-view. In Bin 3, for the experts, both peripheral- and central-views differed from full-view (*p* = 0.002, 0.012, respectively), while central- and peripheral-views did not differ (*p* = 0.129). For the novices, neither the peripheral- or central-view differed from full-view (*p* = 0.663, 0.512, respectively), nor did peripheral- and central-view differ (*p* = 0.344).

In Bins 4 and 5 there were no two-way interaction between group and viewing condition, *F*(2, 56) = 0.54, 0.57, *p* = 0.581, 0.566, respectively, indicating that viewing condition did not have a differential effect on the experts and the novices.

**Eye movement behavior during presentation of test image**

Below we present several analyses to examine the effect of viewing condition on eye-movement behavior.

*Fixation rate*. Figure S2A (left) presents the average fixation rate (fixation count / reaction time) as a function of group and viewing condition. The significant main effect of viewing condition, *F*(2, 54) = 12.61, *p* < 0.001, generalized eta^2^ = 0.06, indicated fewer fixations in central-view relative to full-view (*p* < 0.032) and peripheral-view conditions (*p* = 0.047). There was no difference in fixation count between full-view and peripheral-view conditions (*p* = 0.971). The main effect of group was not significant, *F*(1, 27) = 2.05, *p* = 0.163. Group did not interact with viewing condition, *F*(2, 54) = 0.47 , *p* = 0.629.

*Fixation duration*. Figure S2A (right) presents the average fixation duration as a function of group and viewing condition. The significant main effect of viewing condition, *F*(2, 54) = 21.74 , *p* < 0.001, generalized eta^2^ = 0.09, indicated a shorter average fixation duration in the full-view relative to central-view condition (*p* < 0.001), but not the peripheral-view condition (*p* = 0.080). The average fixation duration was also shorter for the peripheral-view compared to the central-view condition (*p* < 0.001). The main effect of group was not significant, *F*(1, 27) = 1.69 , *p* = 0.204. Group did not interact with viewing condition, *F*(2, 54) = 0.19, *p* = 0.825.


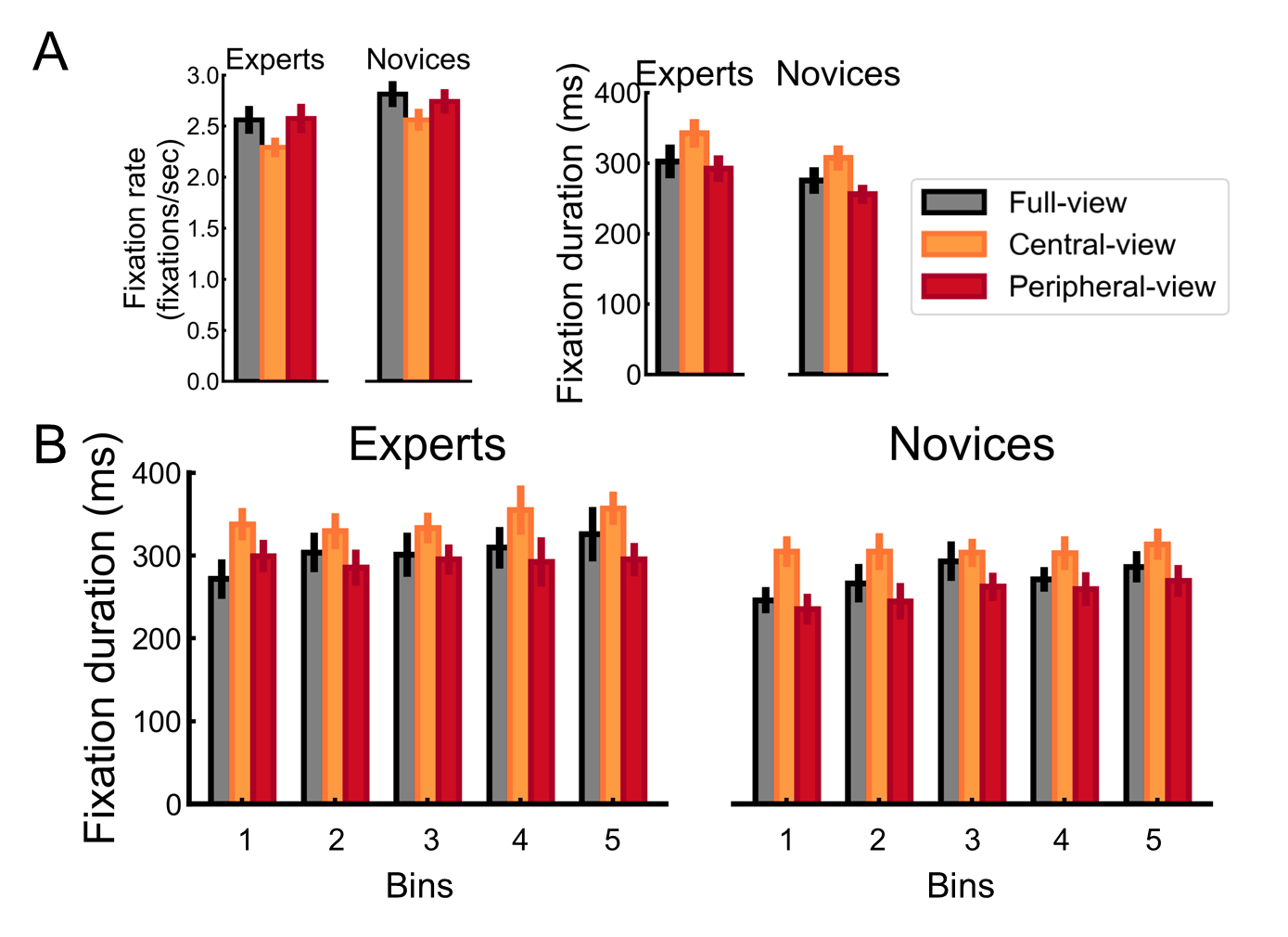


***Figure S2.*** ***A.*** *Fixation rate (left) and average fixation duration (right) as a function of group and viewing condition for correct trials. The rate was calculated by dividing the fixation count in each trial by the response time in that trial.* ***B.*** *Average fixation durations as a function of viewing condition and bin for the novices (left) and experts (right) for correct trials. Bin 1 contains the 20% fastest responses of each participant. Bin 2 contains the next 20% fastest responses, and so on. Error bars represent the SEMs.*

*Distribution analysis of fixation duration.* This analysis specifically tests whether different RTs and viewing conditions are associated with different average fixation behavior. Of particular importance, is the peripheral-view advantage of experts in manual responses associated with longer average fixation durations (e.g., for shifting attention away from fixation)? Figure S2B presents the average fixation duration as a function of group, viewing condition, and quintile bin. The bins were based on reaction times of the manual responses from correct trials. Within each quintile bin, average fixation duration for correct trials were computed. The fixation duration data was analyzed in a mixed-design ANOVA using viewing condition and bin as a within-subjects factor and group as a between-subjects factor. The main effects of viewing condition, *F*(2, 54) = 20.81, *p* < 0.001, generalized eta^2^ = 0.07, and bin, *F*(4, 108) = 3.48, *p* = 0.010, generalized eta^2^ = 0.01, were significant. The main effect of group was not significant, *F*(1, 27) = 2.0, *p* = 0.169. Group did not interact with viewing condition, *F*(2, 54) = 0.19, *p* = 0.828, or with bin, *F*(4, 108) = 0.47, *p* = 0.757. Viewing condition did not interact with bin, *F*(8, 216) = 1.29, *p* = 0.250. The three-way interaction between group, viewing condition, and bin was not significant, *F*(8, 216) = 0.80, *p* = 0.599. Thus, the average fixation duration for experts and novices did not differ as a function of how quickly the participants responded.

*Last fixation*. Here we examine if the experts and novices differ in the ROI they fixate before making a response. This is motivated by the idea that experts and novices may differ in how they seek out sufficient perceptual evidence to base their recognition judgement on. This was examined in the following way: (1) separately for each participant and viewing condition, counting the frequency that the fixation ended in each ROI, (2) computing the proportion of last fixation in different ROIs, by diving the fixation frequency by the total number of trials (i.e., one last fixation per trial), (3) computing a group average by averaging across participants. The data was analyzed in a 2 (group) x 3 (viewing condition) x 5 (ROI) ANOVA with group as a between-subjects factor and viewing condition and ROI as within-subjects factors.

Here we report only the critical statistical outputs relating to group differences. The results are shown in Figure S3. There was no three-way interaction between group, viewing condition and ROI, *F*(8, 216) = 0.26, *p* = 0.979. However, group interacted with ROI, *F*(4, 108) = 3.71, *p* < 0.001, generalized eta^2^ = 0.08. Post-hoc comparisons revealed that this was driven by disproportionately more fixation to the wings for novices than experts, before their response (*m_diff* = 11.72%, *p* = 0.016; all other non-significant *p*s > 0.087). This is consistent with the notion that novices fixate more of an image to acquire sufficient perceptual evidence for making a subordinate category judgement.


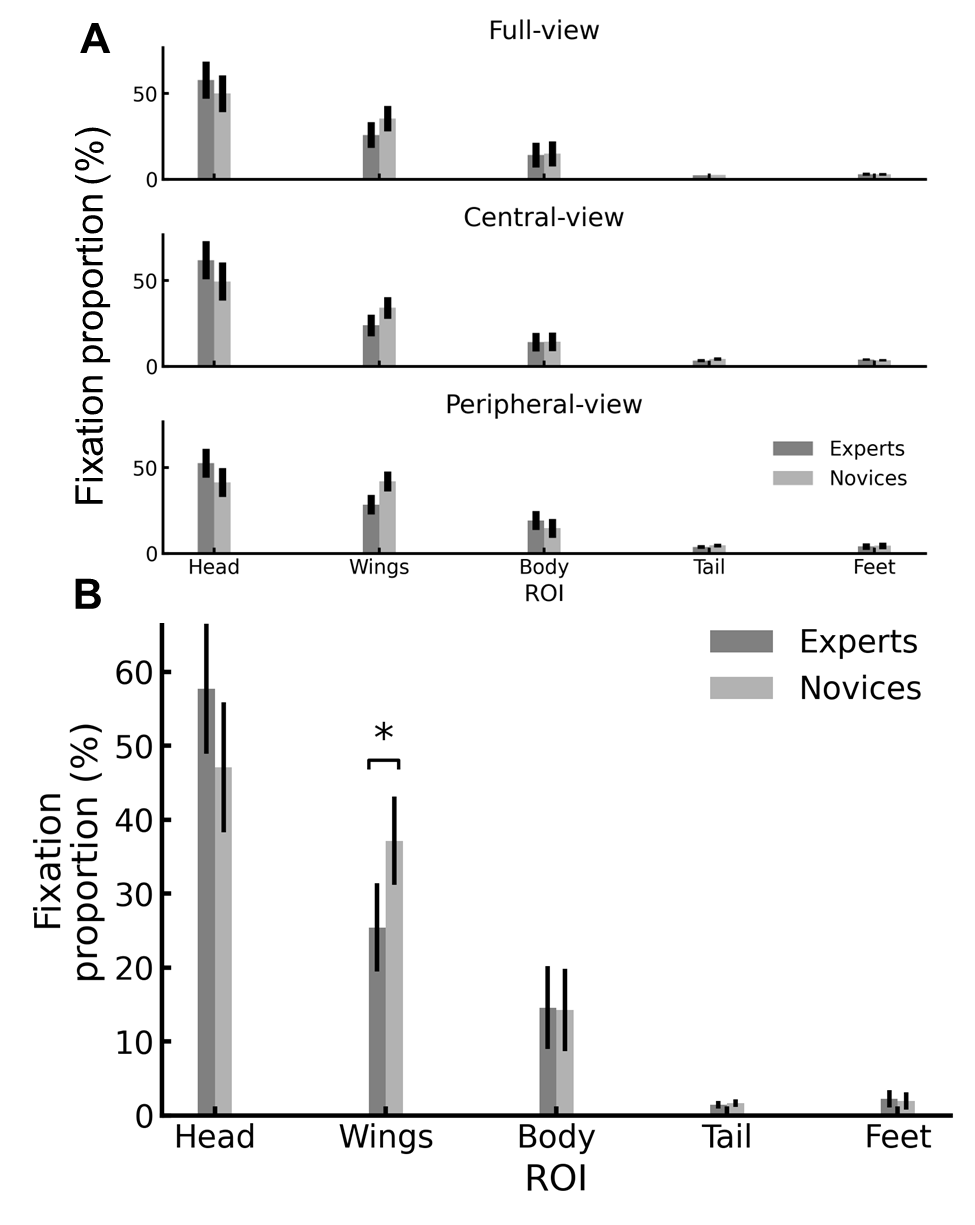


***Figure S3.*** *Fixation proportion across ROIs for the last fixation before the response.* ***A.*** *Fixation proportion as a function of viewing condition, group and ROI.* ***B.*** *Fixation proportion as a function of group and ROI. Proportion is computed by dividing the frequency of fixations in a given ROI by the total fixations across the ROIs. Error bars represent 95% CIs. * p = .016.*

References

1. Rouder, J. N., Morey, R. D., Verhagen, J., Swagman, A. R., & Wagenmakers, E. J. Bayesian analysis of factorial designs. *Psychological Methods,* **22**, 304 (2017).
2. Rouder, J. N., Morey, R. D., Speckman, P. L., & Province, J. M. Default Bayes factors for ANOVA designs. *Journal of Mathematical Psychology,* **56**, 356–374 (2012).
3. Jeffreys, H. *Theory of probability*. Oxford, UK: *Oxford University Press*, (1961).
4. Wagenmakers, E.-J., Wetzels, R., Borsboom, D., & van der Maas, H. L. J. Why psychologists must change the way they analyze their data: The case of psi: Comment on Bem (2011). Journal of Personality and Social Psychology, **100**, 426–432 (2011).
